# Evaluation of a Home Biomonitoring Autonomous Mobile Robot

**DOI:** 10.1155/2016/9845816

**Published:** 2016-04-24

**Authors:** Enrique Dorronzoro Zubiete, Keigo Nakahata, Nevrez Imamoglu, Masashi Sekine, Guanghao Sun, Isabel Gomez, Wenwei Yu

**Affiliations:** ^1^Graduate School of Engineering, Chiba University, Nishi-Chiba, Chiba, Japan; ^2^Artificial Intelligence Research Center, National Institute of Advanced Industrial Science and Technology (AIST), Tokyo, Japan; ^3^Center for Frontier Medical Engineering, Chiba University, Chiba, Japan; ^4^The University of Electro-Communications, Tokyo, Japan; ^5^University of Seville, 41012 Seville, Spain

## Abstract

Increasing population age demands more services in healthcare domain. It has been shown that mobile robots could be a potential solution to home biomonitoring for the elderly. Through our previous studies, a mobile robot system that is able to track a subject and identify his daily living activities has been developed. However, the system has not been tested in any home living scenarios. In this study we did a series of experiments to investigate the accuracy of activity recognition of the mobile robot in a home living scenario. The daily activities tested in the evaluation experiment include watching TV and sleeping. A dataset recorded by a distributed distance-measuring sensor network was used as a reference to the activity recognition results. It was shown that the accuracy is not consistent for all the activities; that is, mobile robot could achieve a high success rate in some activities but a poor success rate in others. It was found that the observation position of the mobile robot and subject surroundings have high impact on the accuracy of the activity recognition, due to the variability of the home living daily activities and their transitional process. The possibility of improvement of recognition accuracy has been shown too.

## 1. Introduction

As a result of a drop in the fertility rates and longer life expectancies, increasing population age turns to be a significantly serious problem in the world [1, 2].

Older population demands more services in healthcare domain. Home biomonitoring is one of such services, especially as population of the single-living elderly (SLE) is increasing. In the past, these services were provided by family members. Nowadays, because of low birth rates and migrations from rural to urban areas, technology solutions to enable the independent life for SLE are strongly required, which will lead to a reduction in hard work of caregivers, time and costs of travels to clinics or hospitals, and so forth.

There have been many efforts to monitor the activities of daily living. Indirect monitoring focuses on used amount of, or status of use of, basic necessities for everyday life, such as lifeline utilities (e.g., electricity, gas, and water supply), and home electrical appliances (e.g., electric pots) [3]. In direct monitoring, the behavior or activities performed by subjects are measured by a set of sensors and analyzed [4–6].

Generally speaking, indirect monitoring is easy to perform. The indicators for all the lifeline utilities and electrical appliances are ready to be used; however, it only provides indirect information of subjects. On the other hand, direct monitoring can provide direct information, which benefits safety of home monitoring; however, additional hardware and software are needed.

There have been three different approaches to acquire the data from the subject and/or environment:

- fixed sensor network: to monitor subjects and house environment change using fixed sensors distributed in the house environment;
- wearable sensors: to acquire biodata from the subject using wearable miniature sensors;
- mobile sensors: to monitor subjects with mobile robots equipped with a small number of sensors.

Advantages and disadvantages of these approaches shall be situation-dependent; however, they could be compared in terms of spatial and temporal continuity of monitoring, in a general sense. Fixed sensor network approach generally needs a large set of sensors, if it aims at covering all the rooms without any dead angles [7]. In the case of furniture layout changes, additional adjustment may be necessary to avoid dead angles. The wearable sensors approach could be a solution to the cost and maintenance problem; however, the constraints to users or their discomfort are the major issues, which could cause discontinuous monitoring. The use of a small number of sensors settled on an autonomous robot that tracks a subject could reduce the cost and deployment complexity. Another advantage of using a robot over the other approaches is the possibility of moving the sensors to place them at an optimal position and angle for the observation.

Traditionally, robots were used to perform repetitive or hazardous tasks. But recently, as great progress has been made in robotics research and development, robotic application is expanding rapidly from the factory into home environment. The idea to use robots in the AAL (Ambient Assisted Life) domain is not new, too. There have been many studies using robots to bring a better quality of life to the elderly [4, 5, 8].

Depending on the level of assistance to ADL, robots could be grouped into the following classes:

- For Self-Maintenance Activities of Daily Living or ADLs [9]: robots that reduce the need for the elderly to move by bringing desired objects to them.
- For Instrumental Activities of Daily Living or IADLs: robots that provide support for ADL, such as meal preparation, laundry, shopping, telephone use; exoskeletal robotic suits and wheelchairs are examples of this class, too.
- For Enhanced Activities of Daily Living or EADLs [10]: many robots are used for hobbies, social communications, new learning, and so forth.

There have been only few reports about home biomonitoring robots [11]. In one of our previous studies, we developed a home biomonitoring robot system with the aim of monitoring motor function impaired persons (MIPs) and the elderly [12]. The robot system developed is able to perform tasks such as subject tracking and behavior observation and analysis [13].

The evaluation of the system has been performed, showing robust subject tracking and accurate behavior recognition. However, the experiments were done in optimal conditions and for a short period of time. There are factors which may appear in real living scenario that may affect the results of the activity recognition. In order to put the home biomonitoring robotic system towards practical use, it has to be tested in home living scenarios.

In this study we performed a series of experiments to investigate the accuracy of activity recognition of the mobile robot in a home living scenario. The daily activities tested in the evaluation experiment include watching TV, reading the newspaper, sleeping, and washing hands. A dataset recorded by a distributed distance-measuring sensor network, synchronized with the robot system with a standardized protocol, was used as a reference to the activity recognition results.

The rest of the paper is organized as follows.  Section 2 describes the system architecture of the biomonitoring robot system. In Section 3 we describe the scenario and experiments used for the evaluation. Experimental results and discussions are given in Section 4, and, finally, concluding remarks are stated in Section 5.

## 2. System Architecture

In this section, a general outline of the robot system for subject tracking and activity recognition and a distance-measuring sensor network used to provide the reference data for the recognized activities will be given, for the purpose of improving readability.

### 2.1. The Autonomous Biomonitoring Robot

**Figure 1:**
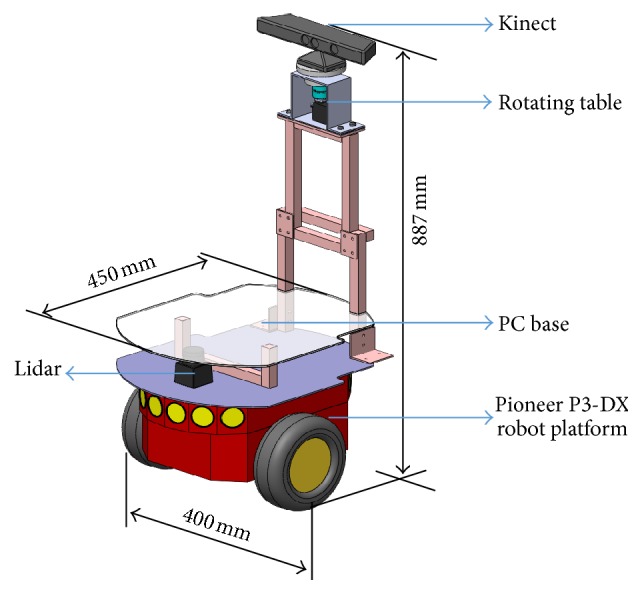
Home biomonitoring robot system.

The autonomous robot (Figure 1) uses Pioneer P3-DX (Adept MobileRobots) as its platform. It includes a Lidar (Light Detection and Ranging) and a Kinect (Microsoft) sensor on a rotation table [14].

The Lidar was used for simultaneous localization and mapping (SLAM), while providing data about the obstacles in the environment. The Kinect sensor is used to detect and track the subject. The rotation table enables the robot to observe the subject while moving forward along with the subject.

In one of our previous studies, an algorithm was proposed and implemented to integrate local 3D observation from the Kinect sensor and global 2D map made from Lidar sensor data to detect and track novelties, as a top-down approach without the necessity of large amount of training data. This solution has proven to have more than 99.00% detection and tracking accuracy in testing datasets [13].

Moreover, the system is able to identify 6 different basic activities: standing, walking, bending, sitting, lying down, and falling. The activity recognition was accomplished using features such as the height-and-width ratio, height change rate, and speed, extracted from human body contour. A state machine based classifier was then employed to classify the features of the activity performed by the subject [15].

Experiments with three subjects were performed. In those experiments the subjects were required to perform a sequence of activities. The overall correct rate of our human activity recognition of those experiments was 98.6–99.4% [15]. The activity recognition could be further improved by making full use of localization information to deal with partial occlusion [14]. However, in those experiments, the activities were performed in a static and repeated manner; that is, after one activity was carried out repeatedly, at one certain place, another activity was tested. The activity performed in different situations, with activity transition, in a home living scenario was not tested.

Moreover, the control parameters of the system have been empirically explored under several environment changes and subject variation, to establish the optimal control strategy to perform the subject tracking and activity recognition [14].

### 2.2. A Sensor Network

In our experiments we used a distance-measuring sensor network to acquire a reference dataset for corroborating the subject location tracked by the biomonitoring robot system. The sensor network was implemented with a platform which provided a standardized interface and network capability to traditional analog sensors [16]. It also provides plug-and-play capabilities and continuous data transmission of more than 10 sensors.

The sensors model used at the experiments is the sharp gp2d12, a distance-measuring sensor with integrated signal processing and analog voltage output. The sensors were placed in a fixed location while the robot is free to move as the scenario designed for the experiments. The communication between the robot and platform was realized by a wireless connection.

**Figure 2:**
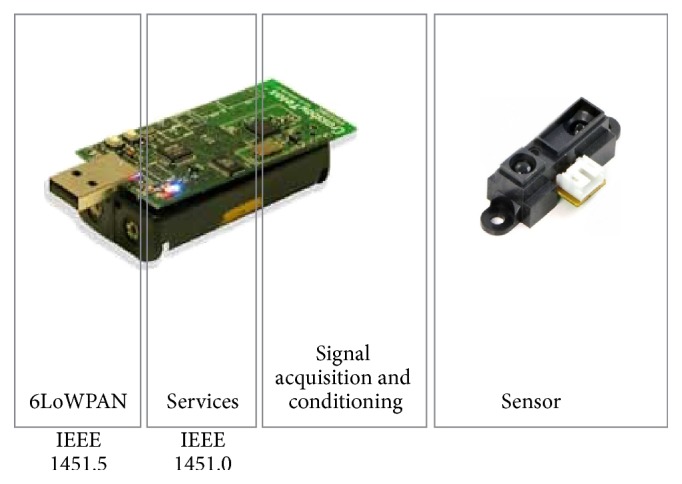
Smart sensor. The sensor is connected to the TelosB mote that provides the signal conditioning and the services and transmission technology defined in the 1451 standards sections 0 and 5, respectively.

The wireless sensor network uses the IEEE 1451 standard. This standard upgrades traditional sensors to a smart status, providing them with a standardized interface and wireless capabilities (Figure 2). Details of the implementation could be found in [16].

## 3. Methodology

A set of experiments were designed to test the robot system in a daily living scenario. The accuracy of the activity recognition was validated by the reference dataset recorded by the distributed distance-measuring sensor network and a video source. The logged data by the robot was synchronized and compared with the recorded video and the sensor dataset. From this comparison the accuracy of the robot system could be determined. The scenario and experiment setting are explained in the following subsections.

### 3.1. Scenario and Activities to Be Recognized

**Figure 3:**
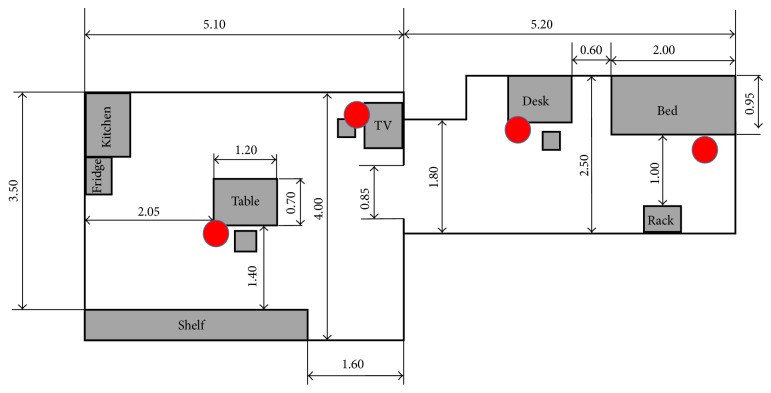
Layout of two rooms for the daily living scenario. Red dots show the position of the distance-measuring sensors.

The layout for the scenario in the experiments is presented in Figure 3. The scenario was tested in a layout with two separated rooms. The main room has one television, one kitchen with a sink and fridge, one table, and one shelf. The second room has one bed and one desk. Distance-measuring sensors were located beside the television, table, desk, and bed (Figure 3).

**Figure 4:**
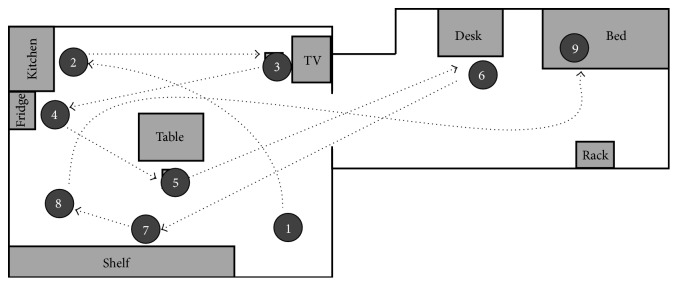
Planned situations and sites. The number represents the order and ID of the scene. The basic activity contained in each situation is presented in Table 1.

In this scenario, nine daily living scenes were planned. The basic activities (such as sitting, bending, and walking) that have been tested for the robot system were included in these scenes, which were scheduled as follows (Figure 4).

At the beginning of the experiment the subject arrives home ①. The robot is waiting at the entrance and it starts tracking the subject. Then, the subject moves towards the kitchen and he washes his hands ②. He walks to the TV, takes a seat, and watches TV ③. After watching TV for a while, he stands up and picks a drink from the fridge ④. When he finishes his drinking, he goes to the table and reads a newspaper ⑤. After reading the paper he moves to his desk and reads a book ⑥. Some minutes later the subject goes to a shelf ⑦ and begins to walk in an open area, as an exercise ⑧. When the exercise is finished he goes to the bed for sleep ⑨.

These scenes include the basic activities that should be recognized by the robot, including walking, standing, bending, sitting, and lying down. The corresponding activities included in each situation are presented in Table 1.

### 3.2. Experimental Tests

Two sets of tests have been performed: activity recognition for scheduled scenes and standing recognition for specific situations.

The first test, activity recognition for scheduled scenes, aims to measure the accuracy of the activity recognition performed in the daily living scenario. The second test aims to investigate how the position of the robot, when tracking the subject, has an impact on the accuracy of the activity recognition process.

Both tests have been performed by two male healthy subjects: (1) subject A: 39 years old, male, 1.76 meters and (2) subject B: 22 years old, male, 1.80 meters.

#### 3.2.1. Activity Recognition for Scheduled Scenes

Two trials were performed. In both trials, the schedule presented in the previous section (Figure 4) was followed. The duration of each activity is shown in Table 1. Each trial was performed by a different subject. During the test, the frames captured by the Kinect on the robot, the activity performed by the subject, the activity recognized by the robot, and the distance-measuring sensor data were recorded. The experiment was filmed by a video camera for further validation.

#### 3.2.2. Standing Recognition for Specific Situations

Currently, the robot decided its observation position according to a minimum-move strategy. This means that for observing an activity the robot position is dependent on its tracking path and no additional movements will be done. However, due to the robot-subject relative position, the accuracy of the activity recognition might be quite different. The aim of this test was to evaluate the impact that the robot position has on the accuracy of the activity recognition system.

Trials were done considering, respectively, the activity of standing, which is much more likely affected by this distance. For these trials the subject stood in front of the robot at distances of 0.5, 1, 1.5, and 2 meters, each position for 2 minutes.

## 4. Results and Discussion

### 4.1. Results

The activity recognition results are summarized in Table 2. Within 43775 frames recorded by Kinect camera of the robot, 33773 frames were matched, which means 77.15% of frames were correctly recognized by the robot.

The recognition accuracy grouped by activity is presented in Table 3. The accuracy for standing, walking, and bending is under 50% while accuracy for sitting and lying down is over 80%.

This information was further broken down in detail into three different tables (Tables 4, 5, and 6). These tables present information about the transition from one scene to another (e.g., ①→②) and the scene itself (i.e., reading the newspaper ③).

During the transition between scenes, the accuracy was drastically decreased (around 51.00%). Scenes ②, ③, and ④ (washing hands, watching TV, and having a drink) also had below-average accuracy (56.99%, 69.21%, and 26.14%, resp.). However, for other scenes ⑤, ⑥, and ⑨ (reading a newspaper, reading a book, and sleeping), high accuracy (93.44%, 81.31%, and 92.42%) was acquired.

The results of scenes ③, ⑤, and ⑥ (watching TV, reading the newspaper, and reading a book) are worth special notice. Despite containing the same basic activity, that is, sitting, the accuracy of the three scenes varies considerably (56.99%, 69.21%, and 93.44%, resp.).

**Figure 5:**
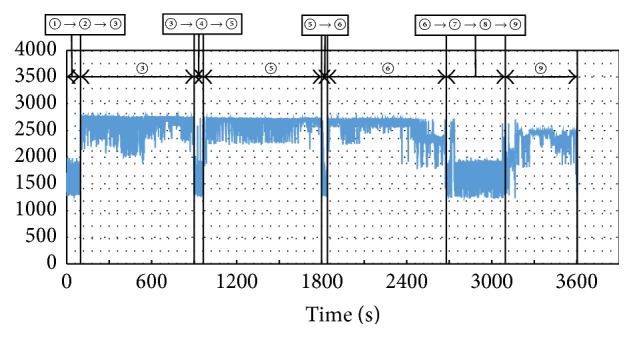
Distance-measuring sensor data for 1-hour experiment. The scene ID included in the graph corresponds with the ones in Table 1.

The distance-measuring sensor data is presented in Figure 5. This data was synchronized with the video recording. The activities have been identified and it could be verified that the high values in the graph corresponded to the scenes in which the sensor was involved (③, ⑤, ⑥, and ⑨). When the subject was in bed, the distance between the sensor and the subject was higher, so it is seen that the values are lower than those of the other activities.

Standing and walking activities presented low accuracy.  Table 7 shows the results of standing trial of test 2. The best result was acquired when the distance was around 1.5 meters. For more than 2 meters or less than 1 meter, the activity could be wrongly recognized as sitting.

### 4.2. Discussion

The evaluation of the system in a home living scenario has been made, using the activity recognition rate and distance-measuring sensor recordings. An average accuracy of 77.15% has been achieved for more than 40.000 frames obtained during the experiments.

**Figure 6:**
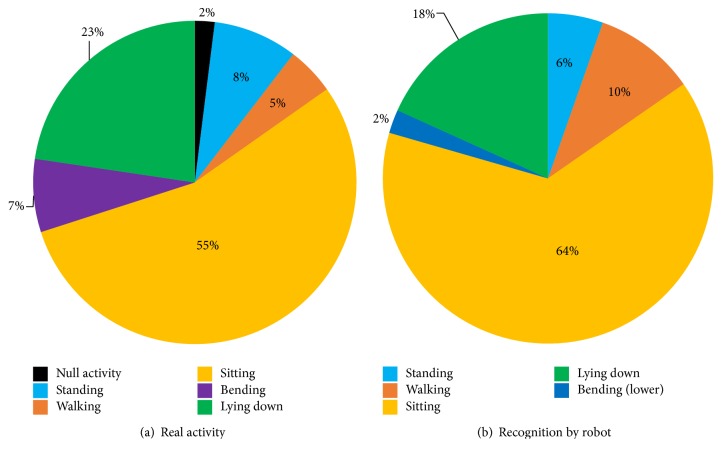
Global ratio of the activities performed by the subject and recognized by the robot.

The results show that this robot system is able to grasp a rough daily life pattern.  Figure 6 presents the ratio activity during the trials, the real one and the one recognized by the robot.

However, the standard deviation for the whole dataset, in terms of different activity, is 29.02%, which means that the accuracy differs considerably between activities.

As shown in Tables 4, 5, and 6, standing and walking activities presented a low recognition rate. The distance between the robot and the subject was an important factor. This factor could be taken into consideration with activity recognition algorithm.

With the actual control policy, the robot moves towards the subject when the distance between both of them is higher than 1.2 meters. During the experiments, when the robot is following a subject and in case the subject stops, the robot stops to keep a distance of 1.2 meters. However, if the subject moves towards the robot, the robot does not move backwards, considering the safety issues. In the daily living scenario, the optimal distance could not be always kept; thus most activity recognition errors occurred in such situations. In several occasions, when the subject was shifting from one scene to another scene, the distance becomes unstable; the activity recognition was likely to fail.

For longer distances, around two meters, accuracy was low too. However, this case should not frequently occur unless obstacles prevented the robot from moving closer to the subject. Actually, this did not happen in test 1, for the scenario and the layout. In the real daily use, if this happens, the robot should inform the subject somehow.

In these two cases, subject above 2 meters or below 1.2 meters, the robot could inform about the impossibility of providing accurate recognition.

There are other activities that present low accuracy results, scenes ③ and ④. In this case, the error in the activity is produced, but the proximity of objects interferes in the extraction of the human body contour.

For instance, we can observe that sitting activity recognition had an average accuracy over 80.24%, but with a standard deviation of 9.89%. While the accuracy keeps high for scenes ⑤ and ⑥, the main problem lies in scene ③, watching TV. This activity has a recognition rate of 69.21%.

The low accuracy results in this specific scene are originated in the process of extracting the human contour, which is critical for the activity recognition. This process extracts a region defined by a radius in the surrounding of tracked point (located in the subject). The proximity of objects at the same depth compared to the subject prevents the activity recognition algorithm from excluding them from the body contour. This fact alters the height-and-width ratio of the features extracted from the human contour leading to wrong activity recognition.

**Figure 7:**
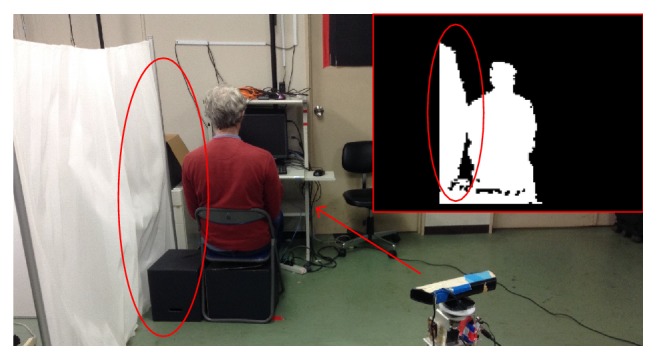
The box and the wall are at the same depth of the person. The activity recognition algorithm limitations include these two objects as part of the body contour.

Figure 7 illustrates this situation. It presents a snapshot of the subject performing scene ③ and the corresponding contour image generated by the activity recognition algorithm. In this figure it is noticeable that the subject, wall, and box are at the same depth, a fact that has a high impact in the recognition process. The contour image reveals that the wall and the box besides the subject are included as part of the body contour. The inclusion of the wall and box as part of the body contour increases the width of the body contour affecting the activity recognition process providing a wrong output. In the example illustrated in Figure 7, the system recognizes the activity of the subject as “bending” instead of the right one, “sitting.”

This issue can be solved using the Kinect data and the map. For a new environment, before it begins subject monitoring, the robot builds an environmental map through SLAM (simultaneous localization and mapping), identifying obstacles such as wall, bed, and tables, as described in [14].

During the robot monitoring operation, it is possible to analyze, in real-time, the Kinect images and check for every pixel whether its coordinates correspond with the position of an obstacle (wall, fridge, etc.) in the environmental map. In that case, generally, the pixel can be safely removed from the image as it is not part of the tracked subject. In consequence, the accuracy of the recognition process will be improved.

The next steps will address the problems observed during this evaluation. Furthermore, we are working towards an easy and fast configuration, through which the robot does not need too much manual calibration for a new environment. The evaluation of the physiological stress of the users to be tracked will be another major concern. We argue that the acceptance of the robot might be improved with the appearance of the robot and its communication capability, without changing the monitoring function. Since, for prospective users, the lonely living elderly with motor function impairment and/or with cognitive function impairment, it is very important and critical to know whether they are safe or not and their life pattern and rhythm, our ultimate goal is to push the monitoring robot to real use in daily living.

## 5. Conclusions

Mobile robots could be a potential solution to home biomonitoring for the elderly. After analyzing the results of the two trial scenarios presented in this paper, it is clear that high accuracy could not be achieved for all the scenes and there are still challenges to overcome.

For some of the scenes of the trial experiment the monitoring system has proven to have an accuracy over 90%. These results are in the range of other human activity recognition systems, Vigilante 92.6%, Tapia et al. 80.6%, and COSAR 93% among others [17]. Please note that their results were achieved with wearable sensors, attached to and relatively static to human body, but also served as constraints to the human body. Nevertheless, in our work, there were other scenes where the accuracy results have to be improved in order to reach acceptable values.

We have identified the two main reasons that lead to wrong recognition: (1) not respecting the minimum distance to perform activity recognition between robot and subject and (2) the presence of obstacles close to the subject in a similar depth that may interfere with the process of extracting the human contour. Further improvement could be reached by improving the body contour detection algorithm and by employing semantic maps, which provide semantic information for the robot to estimate the activity.

On the other hand, the high accuracy activity recognition in some of the daily activities that have been tested proves that mobile robots can perform activity recognition function and become a real solution for in-home monitoring in the future.

## Figures and Tables

**Table 1:** Scene indicates the action performed by the subject while the basic activity is the activity, included in the scene, that the robot will identify.

ID	Scene	Basic activity	Timeline
1	Returning home	Walking	00:00
2	Washing hands	Bending	00:00–00:01 (1 min)
3	Watching TV	Sitting	00:01–00:15 (15 min)
4	Having a drink	Standing and bending	00:15–00:16 (1 min)
5	Reading the newspaper	Sitting	00:16–00:30 (15 min)
6	Reading a book	Sitting	00:30–00:40 (10 min)
7	Picking something from the shelf	Bending	00:40–00:42 (2 min)
8	Stepping	Walking	00:42–00:50 (8 min)
9	Sleeping	Lying down	00:50–01:00 (10 min)

**Table 2:** Summary of the activity recognition results.

Summary	Totals
Frame	43775
Matched frame	33773
Accuracy	77.15

**Table 3:** Recognition accuracy grouped by activity.

	Standing	Walking	Bending	Sitting	Lying down
Frame	1610	4362	995	28052	8000
Matched frame	740	1655	448	22792	7394
Accuracy (%)	45.96	37.94	45.02	81.24	92.42
Standard deviation	31.77	16.2	20.1	9.89	0

**Table 4:** Activity recognition of scene transition phases (1).

Activity	① → ②		②	② → ③		③	③ → ④		④	
	Standing	Walking	Bending	Standing	Walking	Sitting	Standing	Walking	Standing	Bending
Frame	37	157	393	10	120	9872	38	173	80	477
Matched frame	7	86	224	0	65	6833	31	50	11	205
Accuracy (%)	18.91	54.77	56.99	0	54.16	69.21	81.57	28.90	13.75	42.97

**Table 5:** Activity recognition of scene transition phases (2).

Activity	④ → ⑤		⑤	⑤ → ⑥		⑥	⑥ → ⑦ → ⑧			
	Standing	Walking	Sitting	Standing	Walking	Sitting	Standing	Walking	Bending	Bending
Frame	15	138	9691	400	266	8489	544	281	58	26
Matched frame	6	34	9056	306	162	6903	227	185	9	8
Accuracy (%)	40.00	24.63	93.44	76.50	60.90	81.31	41.72	65.83	15.51	30.76

**Table 6:** Activity recognition of scene transition phases (3).

Activity	⑧		⑧ → ⑥ → ⑨				⑨
	Standing	Walking	Standing	Walking	Bending	Bending	Lying down
Frame	756	2631	486	596	16	25	8000
Matched frame	744	707	152	366	2	0	7394
Accuracy (%)	98.41	26.87	31.27	61.40	12.50	0	92.42

**Table 7:** Activity recognition of standing trial of test 2.

	0.5 m	1.0 m	1.5 m	2.0 m
Frame	1400	1400	1400	1400
Matched frame	0	947	1354	416
Accuracy (%)	0.00	67.64	96.71	29.71

## References

[1] Administration for Community Living (2014). *A Profile of Older Americans: 2014*.

[2] European Commission (2015). *The Ageing Report: Underlying Assumptions and Projection Methodologies*.

[3] Fleury A., Noury N., Vacher M. Supervised classification of activities of daily living in health smart homes using SVM.

[4] Ogawa M., Ochiai S., Shoji K., Nishihara M., Togawa T. An attempt of monitoring daily activities at home.

[5] Barger T. S., Brown D. E., Alwan M. (2005). Health-status monitoring through analysis of behavioral patterns. *IEEE Transactions on Systems, Man, and Cybernetics Part A:Systems and Humans.*.

[6] Suryadevara N. K., Mukhopadhyay S. C. (2014). Determining wellness through an ambient assisted living environment. *IEEE Intelligent Systems*.

[7] Snidaro L., Foresti G. L. A multi-camera approach to sensor evaluation in video surveillance.

[8] Huete A. J., Victores J. G., Martínez S., Giménez A., Balaguer C. (2012). Personal autonomy rehabilitation in home environments by a portable assistive robot. *IEEE Transactions on Systems, Man and Cybernetics Part C: Applications and Reviews*.

[9] Lawton M. P., Brody E. M. (1969). Assessment of older people: self-maintaining and instrumental activities of daily living. *Gerontologist*.

[10] Rogers W. A., Meyer B., Walker N., Fisk A. D. (1998). Functional limitations to daily living tasks in the aged: a focus group analysis. *Human Factors*.

[11] Bedaf S., Gelderblom G. J., De Witte L. (2015). Overview and categorization of robots supporting independent living of elderly people: what activities do they support and how far have they developed. *Assistive Technology*.

[12] Myagmarbayar N., Yuki Y., Imamoglu N., Gonzalez J., Otake M., Yu W. Human body contour data based activity recognition.

[13] Imamoglu N., Dorronzoro E., Sekine M., Kita K., Yu W. (2014). Top-down spatial attention for visual search: novelty detection-tracking using spatial memory with a mobile robot. *Advances in Image and Video Processing*.

[14] Imamoglu N., Dorronzoro E., Wei Z. (2014). Development of robust behaviour recognition for an at-home biomonitoring robot with assistance of subject localization and enhanced visual tracking. *The Scientific World Journal*.

[15] Nergui M., Yoshida Y., Imamoglu N., Gonzalez J., Otake M., Yu W. (2013). Human activity recognition using body contour parameters extracted from depth images. *Journal of Medical Imaging and Health Informatics*.

[16] Dorronzoro E., Gómez I., Medina A., Gómez J. (2015). Design and implementation of a prototype with a standardized interface for transducers in ambient assisted living. *Sensors*.

[17] Lara Ó. D., Labrador M. A. (2013). A survey on human activity recognition using wearable sensors. *IEEE Communications Surveys and Tutorials*.

